# Senescence of donor cells impairs fat graft regeneration by suppressing adipogenesis and increasing expression of senescence-associated secretory phenotype factors

**DOI:** 10.1186/s13287-021-02383-w

**Published:** 2021-05-29

**Authors:** Xihang Chen, Jingwei Feng, Qiang Chang, Feng Lu, Yi Yuan

**Affiliations:** Department of Plastic and Cosmetic Surgery, Nanfang Hospital, Southern Medical University, 1838 Guangzhou North Road, Guangzhou, 510515 Guangdong China

**Keywords:** Fat grafting, Adipogenesis, Senescence-associated secretory phenotype, Adipose-derived mesenchymal stem cells, Donor age

## Abstract

**Background:**

Fat grafting has been regarded as a promising approach for regenerative therapy. Given the rapidly aging population, better understanding of the effect of age on fat graft outcomes and the underlying mechanisms is urgently needed.

**Methods:**

C57/BL6 mice [old (O, 18–20-month-old) and young (Y, 4-month-old)] were randomized to four fat graft groups [old-to-old (O-O), young-to-young (Y-Y), old-to-young (O-Y), and young-to-old (Y-O)]. Detailed cellular events before and after grafting were investigated by histological staining, RNA sequencing, and real-time polymerase chain reaction. The adipogenic differentiation potential of adipose-derived mesenchymal stem cells (AD-MSCs) from old or young donors was investigated in vitro. Additionally, adipogenesis of AD-MSCs derived from old recipients was evaluated in the culture supernatant of old or young donor fat tissue.

**Results:**

After 12 weeks, the volume of fat grafts did not significantly differ between the O-O and O-Y groups or between the Y-Y and Y-O groups, but was significantly smaller in the O-O group than in the Y-O group and in the O-Y group than in the Y-Y group. Compared with fat tissue from young mice, senescence-associated secretory phenotype (SASP) factors were upregulated in fat tissue from old mice. Compared with the Y-O group, adipogenesis markers were downregulated in the O-O group, while SASP factors including interleukin (IL)-6, tumor necrosis factor-α, and IL-1β were upregulated. In vitro, AD-MSCs from old donors showed impaired adipogenesis compared with AD-MSCs from young donors. Additionally, compared with the culture supernatant of young donor fat tissue, the culture supernatant of old donor fat tissue significantly decreased adipogenesis of AD-MSCs derived from old recipients, which might be attributable to increased levels of SASP factors.

**Conclusions:**

Age has detrimental effects on fat graft outcomes by suppressing adipogenesis of AD-MSCs and upregulating expression of SASP factors, and fat graft outcomes are more dependent on donor age than on recipient age. Thus, rejuvenating fat grafts from old donors or banking younger adipose tissue for later use may be potential approaches to improve fat graft outcomes in older adults.

**Supplementary Information:**

The online version contains supplementary material available at 10.1186/s13287-021-02383-w.

## Background

Fat grafting has been considered to be a promising regenerative cell-directed therapy and has been successfully used as a regenerative treatment option for many clinical purposes, including breast augmentation and reconstruction, treatment of contour deformities and scars, and wound healing [1–4]. The elderly are increasingly becoming recipients of fat grafting given the desire for a higher quality of life within an aging world population [5]. Emerging evidence suggests that the success of fat grafting is largely dependent on the age of patients [6]. For example, fat grafting is more effective when started at earlier ages for the treatment of Parry–Romberg syndrome or progressive hemifacial atrophy [4]. As plastic surgeons increasingly encounter an aging population, understanding the basic mechanisms of aging combined with how age impacts fat graft outcomes is essential [5].

Adipose tissue is rich in stem cells (adipose-derived mesenchymal stem cells, AD-MSCs), which act as the main player in all types of adipose tissue regeneration, including after fat grafting, by differentiating into adipocytes or vascular endothelial cells and releasing angiogenic growth factors. The “cell replacement theory” states that most adipocytes undergo ischemic apoptosis after fat grafting and are subsequently replaced by regeneration of adipocytes [7, 8]. Furthermore, AD-MSCs in regenerated fat are an admixture of donor and recipient cells, which contribute to regeneration of adipose tissue [9, 10]. However, the age-related effects of AD-MSCs in fat grafts on cell function are not well studied.

During physiological aging, cellular senescence affects multiple tissues, as well as stem cells, which contributes to the loss of functional and regenerative capacity in tissues [11, 12]. This prevents proliferation and differentiation of both somatic and stem cells in a cell-intrinsic manner [13]. Additionally, senescent cells secrete a variety of proteins collectively known as senescence-associated secretory phenotype (SASP) factors. Senescent cells can contribute to loss of tissue function during aging through non-cell-autonomous mechanisms. This can result from SASP factors disrupting homeostasis or possibly through the induction of paracrine senescence in neighboring cells [14, 15]. Aged adipose tissue is reported to release proinflammatory cytokines that impair differentiation of AD-MSCs necessary for regeneration [16]. SASP factors also alter insulin responsiveness, and a decrease in insulin responsiveness promotes lipolysis and production of factors that inhibit adipogenesis [16, 17]. It has long been known that advanced age is negatively correlated with an organism’s reparative and regenerative potential [11, 12], but little information is available about the effect of age on grafted fat tissue remodeling and the underlying mechanisms.

In the present study, we investigated whether age affected fat graft outcomes when grafted into mice of the same age. We also performed a fat cross-grafting experiment between young and old mice to determine whether the effects of age are attributable to the donors or recipients. We performed RNA sequencing (RNA-seq) and real-time polymerase chain reaction (RT-PCR) analysis to investigate age-related changes in the transcriptome of fat grafts in the old-to-old (O-O) and young-to-old (Y-O) groups. Furthermore, to investigate the mechanisms underlying donor age-related differences in fat graft regeneration, the adipogenic differentiation potential of AD-MSCs from old and young donor mice was investigated, and the ability of culture supernatants of old and young adipose tissue to modulate adipogenic differentiation of AD-MSCs from old recipients was determined.

## Methods

### Fat grafting

All experiments were approved by the Nanfang Hospital Animal Ethics Committee Laboratory and were conducted according to the guidelines of the National Health and Medical Research Council of China. Young (4-month-old) and old (18–20-month-old) male C57/BL6 mice weighing 25–30 g were obtained from Southern Medical University, housed in individual cages with a 12-h light/dark cycle, and provided standard food and water ad libitum.

Fat grafting was performed using the following groups (*n* = 6 per group): fat grafts from old-to-old mice (O-O group), fat grafts from young-to-young mice (Y-Y group), fat grafts from old-to-young mice (O-Y group), and fat grafts from young-to-old mice (Y-O group). Fat tissue was harvested from the inguinal fat pads of C57/BL6 donor mice and gently dissected into very small pieces, similar to the size of aspirated fat tissue used for clinical fat injection in humans. A volume of 0.3 mL of prepared adipose tissue was used for fat grafts. An allogenic transplantation (donors and recipients were syngeneic) animal model was used. Each animal (old and young) received one old fat graft on the right side of its dorsum and one young fat graft on the left side of its dorsum. Mice were sacrificed at weeks 1, 4, and 12 after grafting. At the time the animals were sacrificed, grafts were harvested and carefully separated from the surrounding tissue. The water displacement method was used to independently determine the fat graft volume. Fat grafts were immersed in 0.9% saline, and the displaced solution was measured using a graduated cylinder to determine the fat graft volume [18]. Each harvested sample was assessed by different methods.

### Histological, immunohistochemical, and immunofluorescence analyses of fat grafts

Tissue samples were fixed in 4% paraformaldehyde, dehydrated, and embedded in paraffin for staining with hematoxylin and eosin (HE).

Tissue samples were immunohistochemically stained with rabbit anti-mouse histone H2AX phosphorylation (γH2A.X) (1:200, ab26350; Abcam, Cambridge, UK) and rabbit anti-mouse p21 (1:200, ab188224; Abcam) primary antibodies and then with a horseradish peroxidase-conjugated goat anti-rabbit IgG H&L secondary antibody (1:1000, ab205718; Abcam).

After grafting, tissue sample sections were immunofluorescently stained with a rabbit anti-mouse perilipin primary antibody (1:200, ab3526; Abcam), before being washed and labeled with an Alexa Fluor® 594-conjugated goat anti-rabbit IgG secondary antibody (1:1000, ab150080; Abcam). Nuclei were stained with DAPI (1:10000, D9542; Sigma, St. Louis, MO, USA). To quantitate adipogenesis, the percentage of perilipin-positive areas was determined by dividing the positive area by the total area. All images were captured with a microscope (Olympus BX63; Olympus, Tokyo, Japan). Images were analyzed using ImageJ software.

### RNA-seq analysis of fat tissue before and after grafting

To explore the influence of age on fat grafting, RNA was prepared from fat tissue before grafting from old and young mice for RNA-seq analysis (three biological replicates per group). Fat grafts from the O-O and Y-O groups were also collected at 1 week after grafting, and RNA was extracted and prepared for RNA-seq (three biological replicates per group). RNA-seq experiments were performed by Novogene (Beijing, China). Briefly, total RNA was isolated from fat tissue using TRIzol reagent (Invitrogen, CA, USA). Library preparation and transcriptome sequencing were carried out using Illumina HiSeq X Ten (Novogene Bioinformatics Technology Co., Ltd., Beijing, China). Mapping of 150-bp paired-end reads to genes was undertaken using Hisat2 v2.0.5 software, and fragments per kilobase of transcript per million fragments mapped (FPKM) were also analyzed. Differential expression was analyzed using the edgeR R package (3.22.5). p values were adjusted (padj) using the Benjamini and Hochberg method. Gene ontology (GO) analyses were undertaken using the clusterProfiler R package. The hierarchical clustering heat map was generated with the ggplot library.

### RT-PCR analysis

RNA was isolated and quantified before cDNA was synthesized using PrimeScript™ RT Master Mix (TaKaRa, Kyoto, Japan). PCR was performed using a LightCycler 480 Real-time PCR System (Roche, Indianapolis, IN, USA) and SYBR® Premix Ex Taq™ (TaKaRa). Expression levels were calculated using the 2^−ΔΔCt^ method. The following primers were used: *Pparg* (forward, 5′-GAACCTGFATCTCCACCTTATT-3′; reverse, 5′-TGGAAGCCTGATGCTTTATCC-3′); *Tnf* (forward, 5′-CAGCAAGCACTCAACGGAAT-3′; reverse, 5′-CGTCCTCTGAACGACCAACA-3′); *Il1b* (forward, 5′-AGTTGACGGACCCCAAAAG-3′; reverse, 5′-TTTGAAGCTGGATGCTCTCAT -3′); *Il6* (forward, 5′-ACAGAAGGAGTGGCTAAGGA-3′; reverse, 5′-TTTCTGACCACAGTGAGGAA-3′); and *Gapdh* (forward, 5′-AACTTTGGCATTGTGGAAGG-3′; reverse, 5′-CCCTGTTGCTGTAGCCGTAT-3′).

### Cell culture and fat tissue culture

AD-MSCs were isolated from inguinal fat pads of old or young mice using a previously described protocol [19, 20]. In brief, fat tissue was digested with 0.075% collagenase A (Sigma) diluted in phosphate-buffered saline (PBS) for 40 min on a shaker at 37°C. Collagenase digestion was stopped by adding AD-MSC complete medium (MUBMD-90011; Cyagen, Guangzhou, China), which consisted of DMEM supplemented with 10% fetal bovine serum, 1% penicillin–streptomycin, and 1% glutamine. After centrifugation, the supernatant was discarded. Cell pellets were resuspended in AD-MSC complete medium, centrifuged, filtered through a 100-μm cell strainer, and resuspended in AD-MSC complete medium. The cells were maintained at 37°C in a humidified atmosphere containing 5% CO_2_ and 95% air. The medium was changed after 24 h and then every second day. The cells were passaged once after reaching full confluency. Cells at passage 3 were used in this study.

To test the effect of age on adipogenesis of AD-MSCs, AD-MSCs from old or young mice were seeded at a density of 20,000 cells per cm^2^ and incubated in an adipogenic medium (MUBMD-90031; Cyagen), which consisted of DMEM containing 10% fetal bovine serum, 1% penicillin–streptomycin, 0.1 mM ascorbic acid, 1 μΜ dexamethasone, and 0.5 mM 3-isobutyl-1-methylxanthine, for 15 days. The medium was changed every 3 days. The cells were maintained at 37°C in a humidified atmosphere containing 5% CO_2_ and 95% air. After 15 days of culture in the adipogenic medium, perilipin was labeled to identify lipid vacuoles in cells for assessment of adipogenic differentiation.

Samples were processed for tissue culture as previously described [21]. Briefly, excised adipose tissue from old or young mice was collected. After removing vessels and conjunctive tissue, adipose tissue was washed with Krebs–Ringer HEPES and cut into small pieces with sharp scissors. Tissue fragments were placed in 6-well dishes (300–400 mg adipose tissue/well) containing 2.5 mL DMEM supplemented with 0.5% fetal bovine serum (Gibco, Grand Island, NY, USA), 100 U/mL penicillin (Gibco), and 100 μg/mL streptomycin sulfate (Gibco) [21]. Culture supernatants of old and young adipose tissue were collected at day 3 and their paracrine effects were evaluated.

Old AD-MSCs were cultured in a conditioned medium (adipogenic medium supplemented with the culture supernatant of old or young adipose tissue at a 1:1 ratio) for 15 days. The conditioned medium was replaced every 3 days. After 15 days of culture in the conditioned medium, cells were stained for perilipin to detect lipids.

### Assessment of adipogenesis of AD-MSCs

In vitro adipogenesis was evaluated by measuring increases of lipids/triglycerides in cultured differentiated AD-MSCs. Perilipin staining was used to visualize accumulated lipid-rich cytoplasmic vacuoles. Briefly, differentiated AD-MSCs were fixed with 4% paraformaldehyde, rinsed in PBS, and blocked for 1 h with Tris-buffered saline (TBS, pH 7.4) containing 1% bovine serum albumin and 0.01% Triton X-100. Next, samples were incubated overnight at 4°C with a rabbit anti-mouse perilipin primary antibody (1:100, ab3526; Abcam), rinsed extensively with TBS, and incubated with an Alexa Fluor® 594-conjugated goat anti-rabbit IgG secondary antibody (1:1000, ab150080; Abcam) for 1 h at room temperature. Finally, samples were rinsed in PBS and stained with DAPI (Sigma). All images were captured with a microscope (Olympus BX63) using the same laser intensity and detection sensitivity. The area occupied by lipid vacuoles was analyzed with ImageJ based on the sections stained for perilipin.

### Characterization of AD-MSCs via flow cytometry

To analyze the expression of typical cell surface markers, cultured AD-MSCs at passage 3 were treated with the following anti-mouse conjugated antibodies on ice for 30 min in the dark: PE-CD73, FITC-CD90, PE-CD29, APC-CD31, FITC-CD45, and APC-CD34. Cell suspensions not labeled with antibodies served as controls. Cells were washed twice and resuspended in 300 μL of PBS before analysis. All antibodies were purchased from BD Biosciences (San Diego, CA, USA). Flow cytometry was performed with a flow cytometer (BD FACS Vantage SE, BD Biosciences).

### Western blot analysis of AD-MSCs in vitro

Total cell lysates of cultured AD-MSCs were prepared using M-PER Mammalian Protein Extraction Reagent (Thermo Fisher Scientific, Waltham, MA, USA). Primary antibodies against p21 (1:1000, ab188224; Abcam) and γH2A.X (1:1000, ab26350; Abcam) were used. After incubation with secondary antibodies, immunocomplexes were detected with a WesternBreeze Chemiluminescent Detection Kit (WB7108; Thermo Fisher Scientific). β-actin was used as an internal control.

### Senescence-associated β-galactosidase (SA-β-gal) staining

To detect cellular senescence, a SA-β-gal staining kit was used (Cell Signaling Technology, Boston, MA, USA). Briefly, cells were seeded into 12-well plates at a density of 5 × 10^3^ cells/well and incubated with freshly prepared β-gal staining solution for 60 min at 37°C in the absence of CO_2_. AD-MSCs were washed with water, and blue labeling (i.e., senescent cells) was observed under a microscope. SA-β-gal-positive cells (blue) were counted under a microscope and expressed as the percentage of total cells.

### MILLIPLEX® MAP assays

The levels of the following mouse cytokines in adipose tissue culture media were evaluated by multiplex analysis: granulocyte-macrophage colony-stimulating factor (GM-CSF), interferon γ (IFNγ), interleukin (IL)-1α, IL-1β, IL-2, IL-4, IL-5, IL-6, IL-7, IL-10, IL-12 (p70), IL-13, IL-17A, chemokine (C-X-C motif) ligand 1 (Cxcl1), Cxcl5, monocyte chemoattractant protein (MCP)-1, macrophage inflammatory protein (MIP)-2, and tumor necrosis factor (TNF)-α. The assay was performed using the MILLIPLEX magnetic bead panel (Millipore Corporation, Billerica, MA, USA) in accordance with the manufacturer’s instructions. Briefly, 25 μL of adipose tissue culture medium was incubated with 25 μL of fluorescent magnetic beads coated with antibodies in a 96-well plate overnight at 4°C. Plates were then washed and incubated with detection antibodies (1 h at room temperature) and the reporter streptavidin–phycoerythrin (30 min at room temperature). Finally, samples were run on the Luminex 100/200 system and data were collected using Luminex xPONENT® software (v. 3.1). The median fluorescence intensities (MFIs) of cytokine/chemokines were analyzed using MasterPlex® QT software (v1.1) and the Luminex 200 analyzer (Luminex Corporation, Austin, TX, USA). The MFI data were saved and analyzed using a five-parameter logistic for calculating analyte concentrations in samples. Triplicate tests were performed for each sample.

### Statistical analysis

Data analysis was performed using GraphPad Prism statistical software. Data are presented as mean ± standard error of mean (SEM) unless otherwise stated. The numbers of technical and experimental replicates for each experiment are provided in the figure legends. The independent samples *t*-test or a two-way ANOVA with Bonferroni’s post hoc analysis was used where appropriate. *p* values < 0.05 were considered statistically significant. Differential gene expression was considered significant at padj < 0.05. For term enrichment in GO analysis, the level of significance was set at a padj < 0.01.

## Results

### Older age increases senescence in fat tissue

**Fig. 1:**
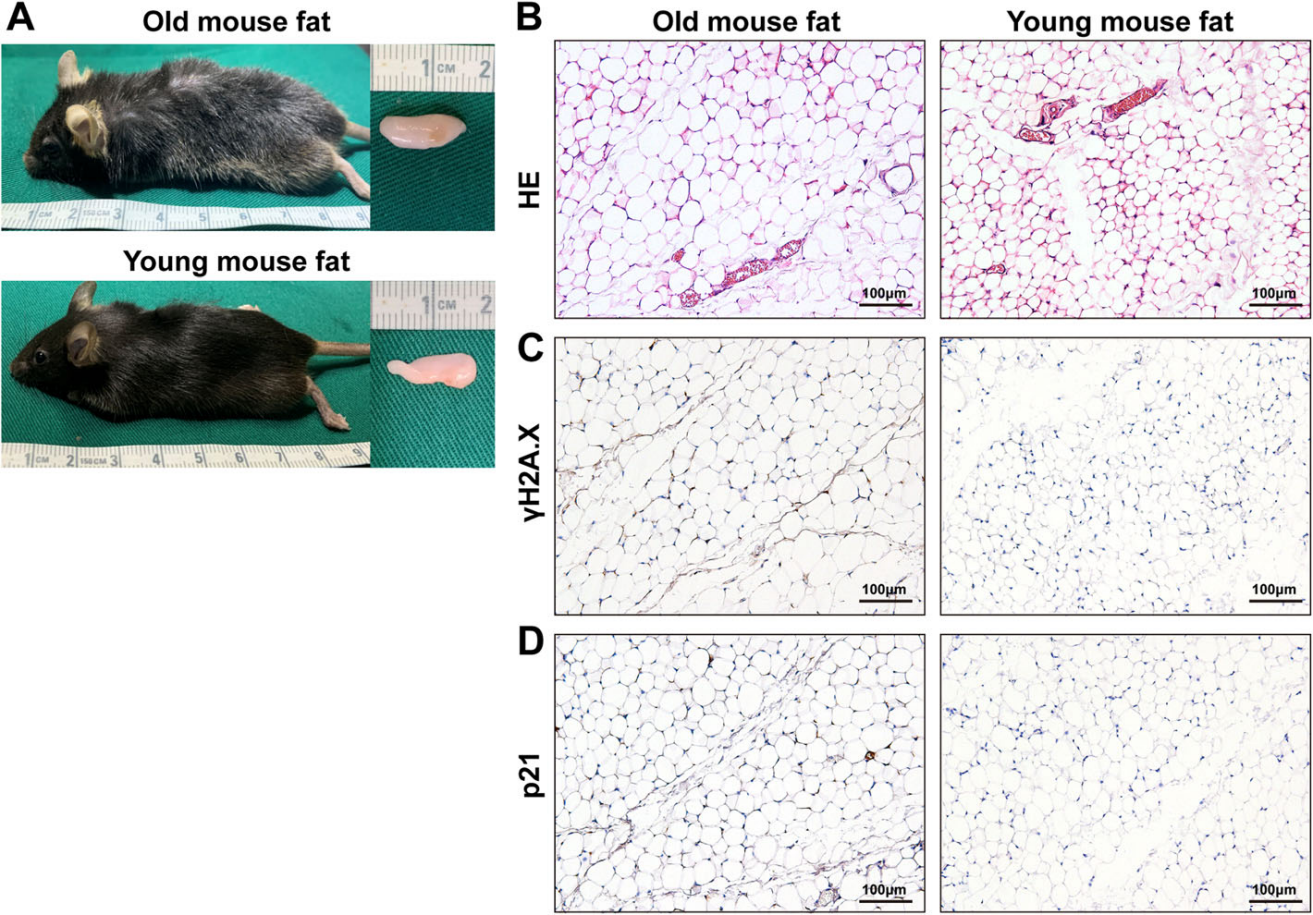
Identification of fat tissue from old mice. **a** Macroscopic views, **b** histologic evaluation, and immunohistochemistry of **c** γH2A.X and **d** p21 of fat tissue from old and young mice

Older fat tissue exhibited hallmark features of senescence including a more uneven texture and darker color (Fig. 1a). Histological analysis showed that fat tissue in old mice displayed a loose tissue structure and larger adipocytes than that observed in young fat tissue (Fig. 1b).

Fat tissue from old mice exhibited increased expression of the age-related markers γH2A.X (Fig. 1c) and p21 (Fig. 1d).

### Age of donor has a significant effect on fat graft outcomes

**Fig. 2:**
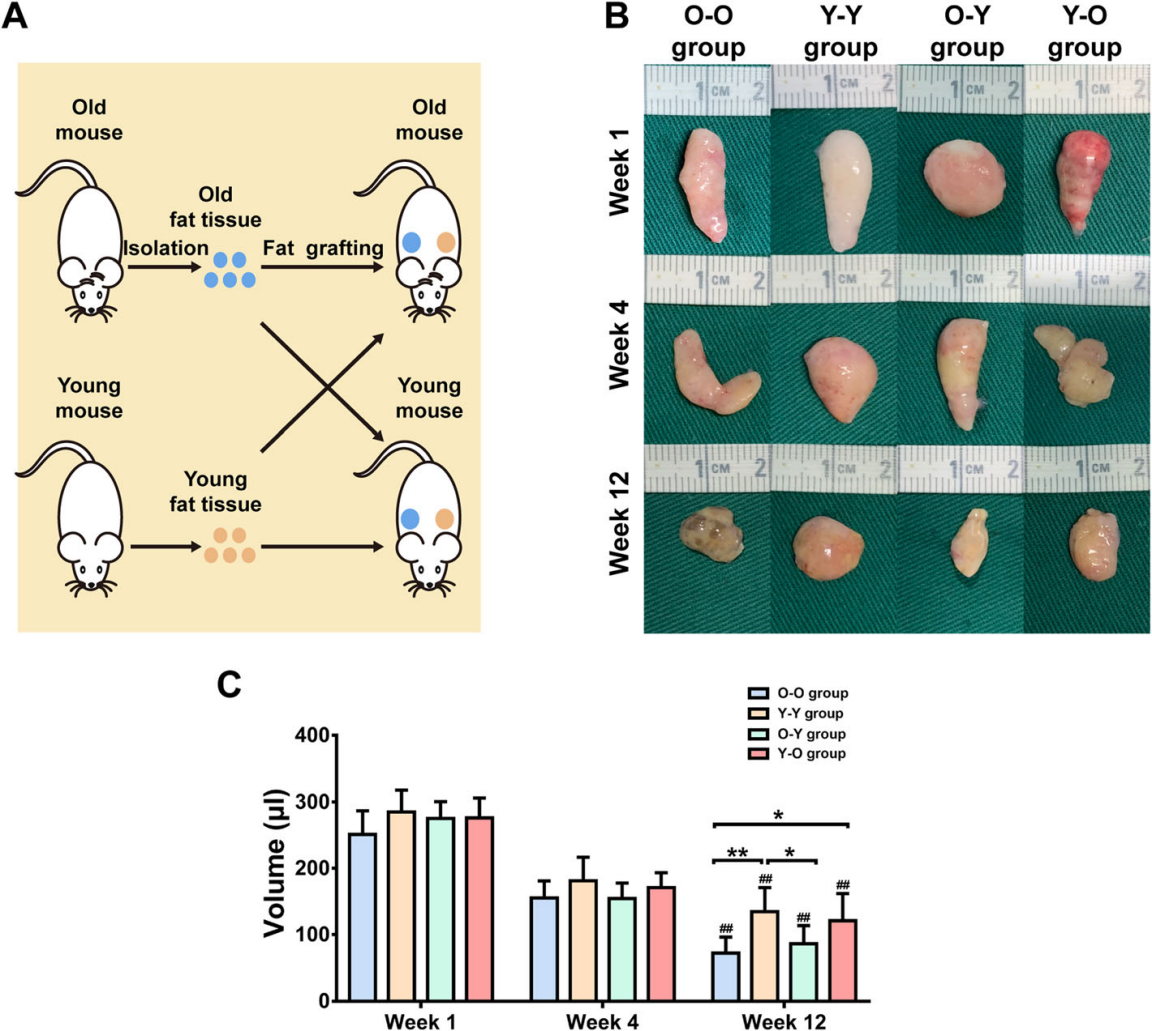
Experimental design and graft outcomes in the four groups. **a** Experimental design. **b** Macroscopic views of harvested tissue from the O-O, Y-Y, O-Y, and Y-O groups. **c** Volumetric data indicated that the graft volume changed over time after injection, decreasing from the initial volume of 300 μL. ^##^*p* < 0.01 vs. data from the same group at week 1; **p < 0.01 and *p < 0.05 vs. data from different groups. Data are presented as the mean ± SD (*n* = 6 per group)

**Fig. 3:**
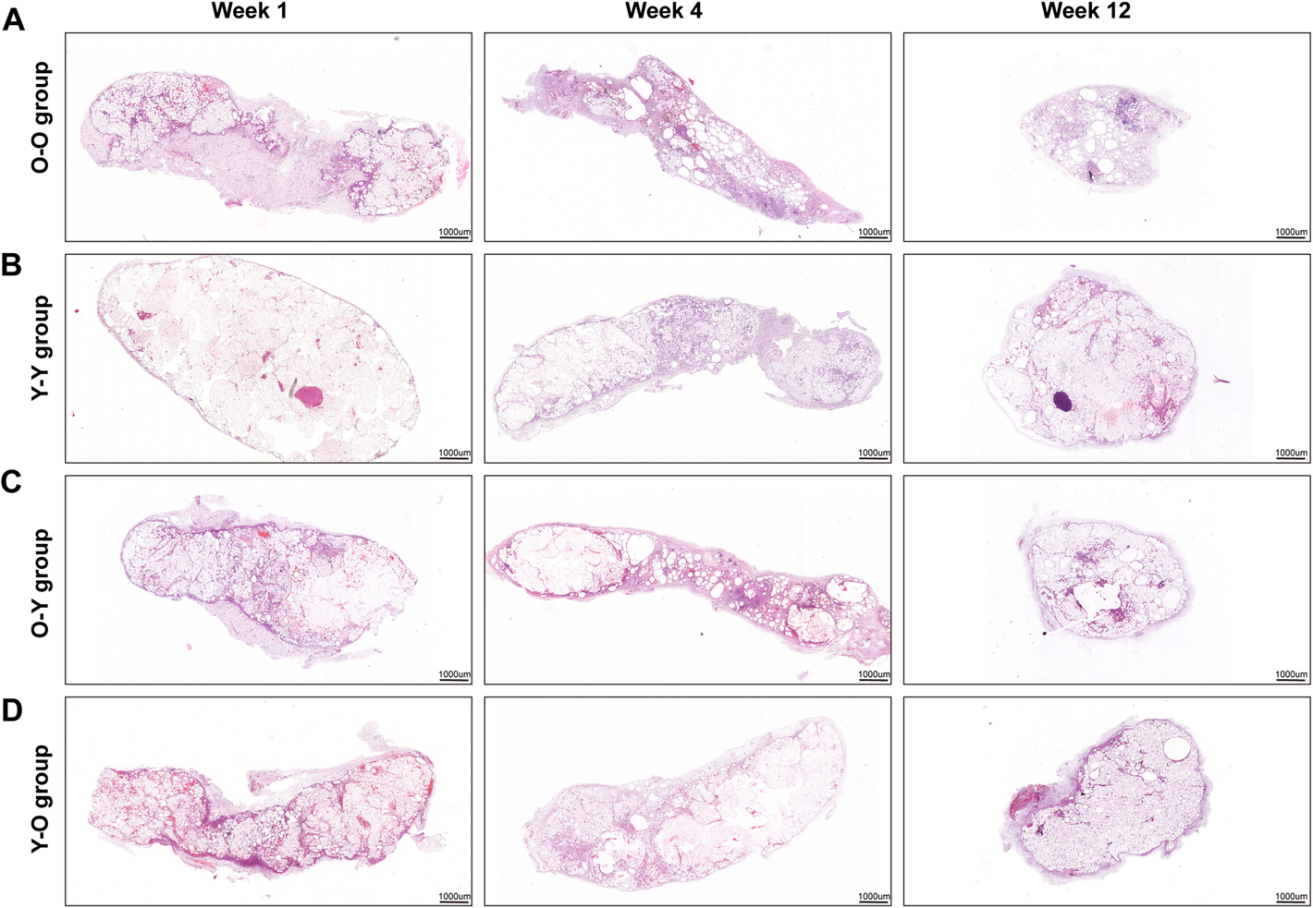
Histologic changes in grafts over time. Histologic evaluation of the fat grafts from the **a** O-O, **b** Y-Y, **c** O-Y, and **d** Y-O groups at weeks 1, 4, and 12. The O-O and O-Y groups exhibited numerous inflammatory cells, large oil droplets, and necrotic nodules in the interior zone of the graft. By contrast, grafts in the Y-Y and Y-O groups had mature, vascularized fat tissue at week 12

**Fig. 4:**
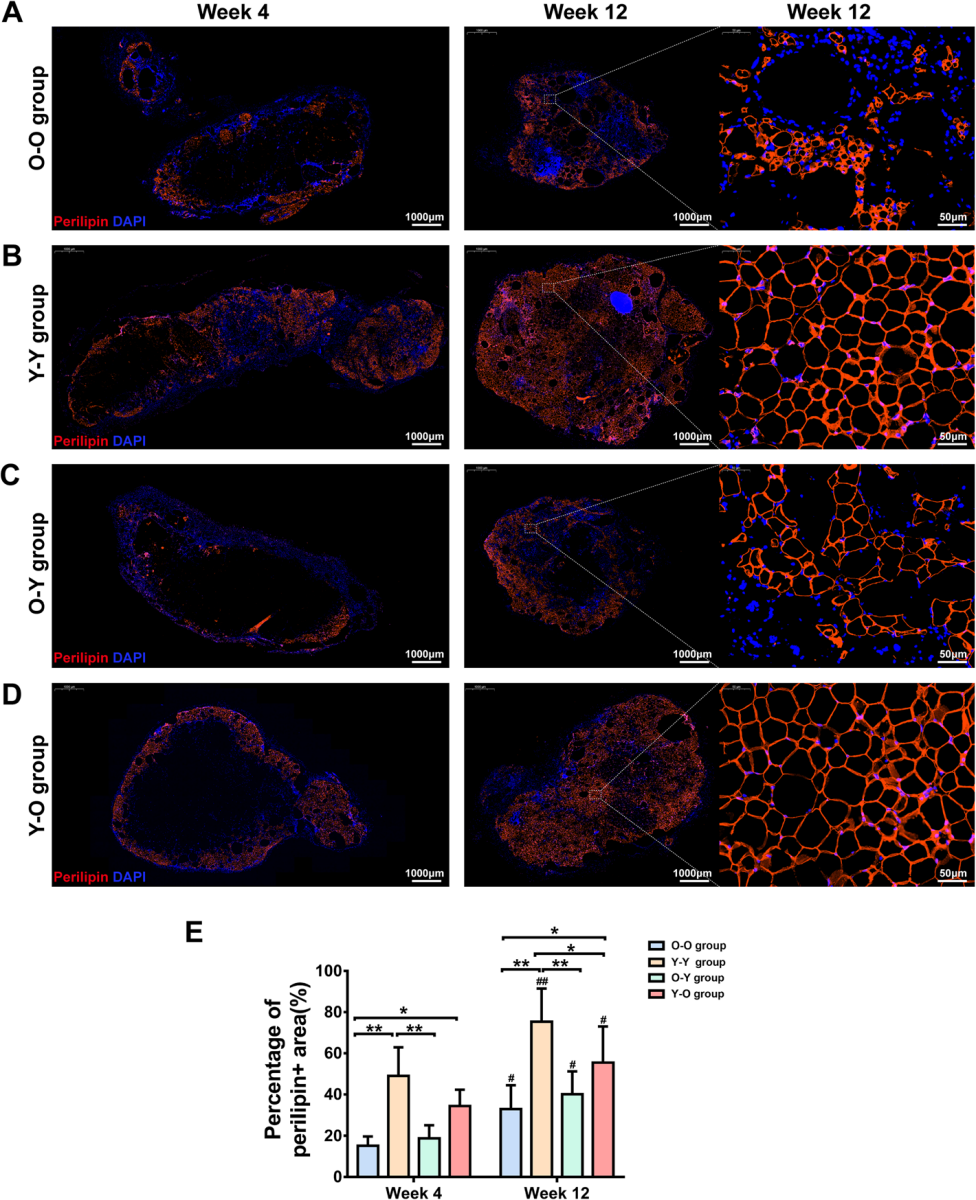
Immunohistochemistry of perilipin-positive adipocytes. Fat graft samples from the **a** O-O, **b** Y-Y, **c** O-Y, and **d** Y-O groups at weeks 4 and 12 were stained for perilipin. Fat tissue in the O-O and O-Y groups appeared to be less integrated and displayed immature adipocyte with a smaller size than fat tissue in the Y-Y and Y-O groups at week 12. **e** Percentage of perilipin-positive areas indicated that all four groups presented fat regeneration over time because perilipin-positive areas in graft samples increased from week 4 to 12. ^**##**^*p* < 0.01 and ^**#**^*p* < 0.05 vs. data from the same group at week 4. **p < 0.01 and *p < 0.05 versus data from different groups. Data are presented as the mean ± SD (n = 6 per group)

To assess the effect of donor age on fat graft outcomes, fat tissue from old and young donors was placed into old recipients (O-O and Y-O) (Fig. 2a). The fat graft in the O-O group presented poor surface vascularization and visible oil cysts; however, the fat graft in the Y-O group appeared to be normal (Fig. 2b). The fat volume was significantly smaller in the O-O group than in the Y-O group at week 12 (72.5 ± 23.61 μL vs. 120.83 ± 40.79 μL; *p* < 0.05) (Fig. 2c). Histological analysis showed that a small number of oil cysts were observed in the central zone of fat grafts from the O-O group at week 1, and the number and volume of these cysts increased over time. Some large oil cysts had not been completely absorbed by week 12 (Fig. 3a). In contrast with the incomplete fat tissue structure observed in the O-O group, grafts from the Y-O group presented a relatively complete fat structure with larger mature blood vessels, except for some scattered small oil cysts at week 12 (Fig. 3d). Moreover, small, immature adipocytes and large perilipin-negative regions existed in the O-O group (Fig. 4a), in contrast with the mature adipocytes found in the Y-O group at week 12 (Fig. 4d). Although perilipin-positive areas were increased from week 4 to week 12 in both groups, they were significantly smaller in grafts from the O-O group than from the Y-O group at weeks 4 and 12 (Fig. 4e).

When older age donor tissue was placed into a young recipient (O-Y), grafts presented as a small mass with a yellow–gray color. By contrast, grafts from the Y-Y group showed a normal appearance with a bright pink color (Fig. 2b). In addition, fat volume was significantly smaller in the O-Y group than in the Y-Y group (85.83 ± 27.64 μL vs. 134.17 ± 36.66 μL; *p* < 0.05) (Fig. 2c). Histological analysis showed that large necrotic areas and a large amount of oil cysts were observed in the interior zone of grafts from the O-Y group from week 1 to week 12 (Fig. 3c). By contrast, although a small number of oil cysts was also observed in grafts from the Y-Y group from week 1 to week 12, most necrotic areas and oil cysts had been replaced by mature adipocytes at week 12 (Fig. 3b). In addition, adipogenesis in the O-Y group was incomplete, as characterized by smaller adipocytes and more residual undifferentiated regions compared with the Y-Y group (Fig. 4b, c). Perilipin-positive areas were increased from week 4 to 12 in both groups, but were significantly smaller in grafts from the O-Y group than from the Y-Y group at weeks 4 and 12 (Fig. 4e).

### Age of recipient has a minimal effect on fat graft outcomes

To determine the impact of recipient age on fat graft outcomes, fat tissue from old mice was grafted into old or young recipients (O-O and O-Y) (Fig. 2a). Age difference in the recipients did not significantly affect fat graft appearance or volume. At 12 weeks after grafting, the grafts displayed a grayish appearance (Fig. 2b), and the volume of grafts shrank considerably in both groups (Fig. 2c). Incomplete fat tissue structure (Fig. 3a, c) and low levels of perilipin-positive areas were also observed in both groups at week 12 (Fig. 4e).

When lipoaspirate was taken from young mice and grafted into old or young mice (Y-O and Y-Y), both groups exhibited normal tissue appearance (Fig. 2b). Fat volume (Fig. 2c) and tissue structure (Fig. 3b, d) of grafts did not differ significantly between the groups at week 12. Perilipin-positive areas were slightly larger in younger recipients at week 12; however, this effect was not significant (Fig. 4e).

### SASP factors are upregulated in fat tissue from old mice

Overall, the above findings suggested that fat grafts from old donors led to lower graft retention compared with those from young donors, which was associated with impaired fat regeneration. To gain more insight into the deleterious effects of donor age on fat graft regeneration, RNA was prepared from fat tissue harvested from old and young mice for RNA-seq analysis.

**Fig. 5:**
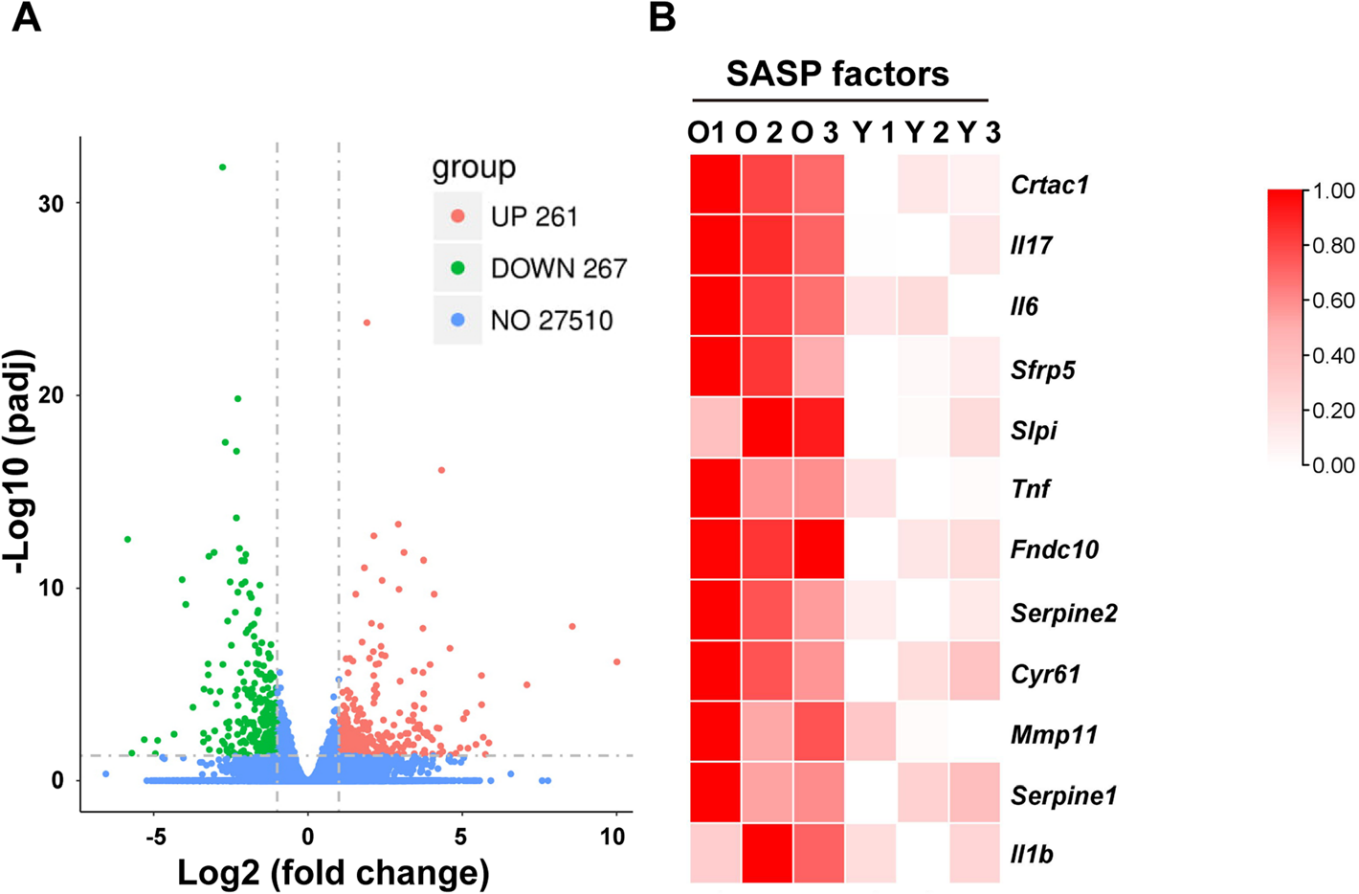
Transcriptomic analysis of fat tissue from old and young mice. **a** Volcano plot of DEGs in fat tissue from old and young mice. –log10 padj and log2 (fold change) values are shown. **b** Heat map showing the expression levels of selected genes associated with SASP factors

Among 27,510 detected genes, 261 were significantly upregulated and 267 were downregulated (padj < 0.05 and log2 (fold change) > 1) in fat tissue harvested from old mice compared with grafts from young mice (Fig. 5a and Supplemental Table 1). Notably, among the 261 significantly upregulated genes, a group of genes was related to SASP factors including *Crtac1*, *Il17*, *Il6*, *Sfrp5*, *Slpi*, *Tnf*, *Fndc10*, *Serpine2*, *Cyr61*, *Mmp11*, *Serpine1*, and *Il1b* (Fig. 5b).

### Adipogenesis is suppressed in grafts from old donors at week 1

To gain more insight into the deleterious effects of donor age on fat graft regeneration after grafting, RNA was prepared from fat grafts of mice at 1 week after grafting in the O-O and Y-O groups for RNA-seq analysis.

**Fig. 6:**
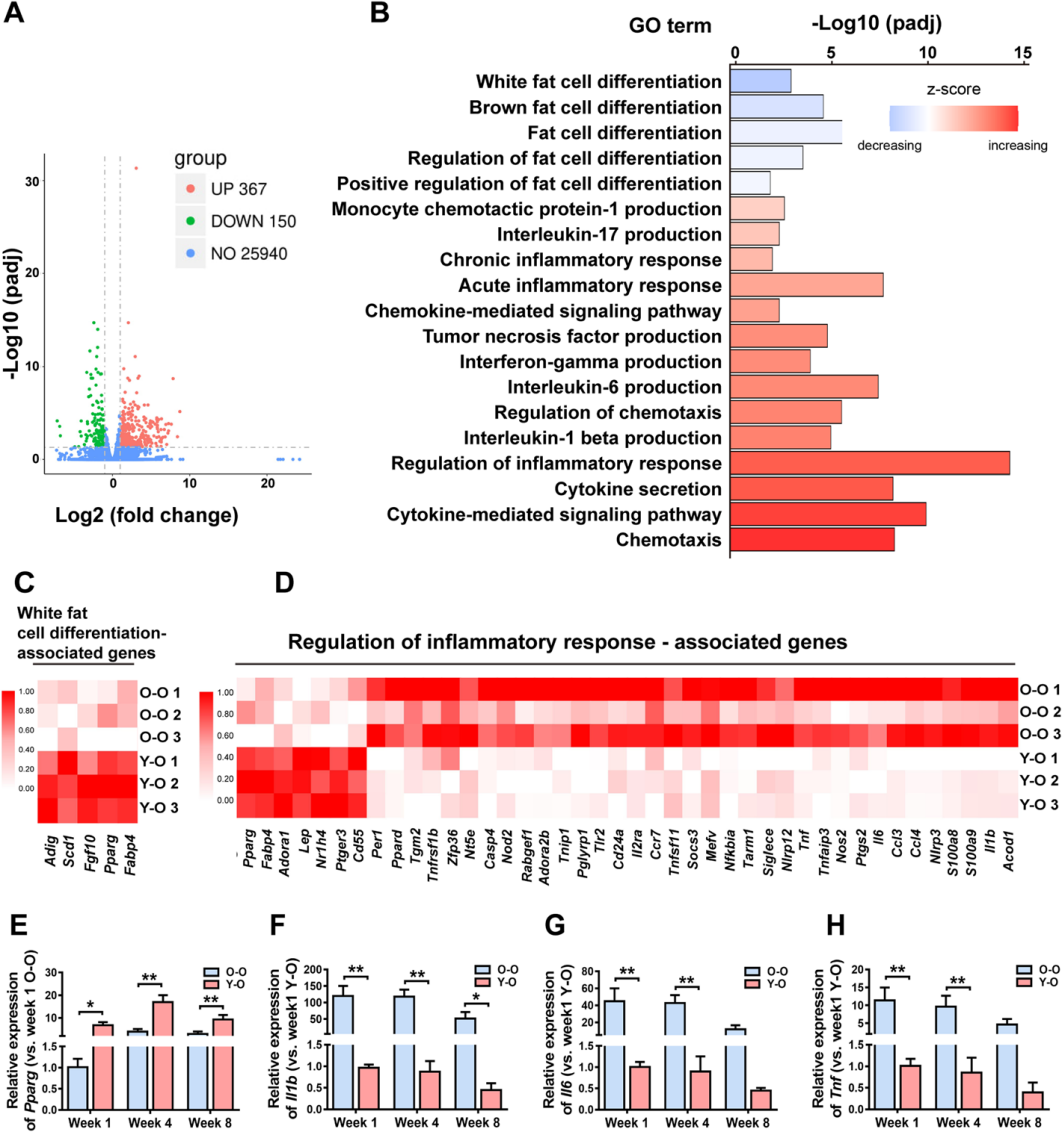
Transcriptomic analysis of fat grafts from the O-O and Y-O groups. **a** Volcano plot of DEGs between fat grafts at week 1 in the O-O and Y-O groups. –log10 padj and log2 (fold change) values are shown. **b** Enriched GO terms including “adipogenesis” and “tissue inflammation” associated with the DEGs in fat grafts from old donors compared with young donors. To capture the overall tendency toward upregulation or downregulation of each term, a z-score was calculated ($Z=\frac{\left(\mathrm{up}-\mathrm{regulated}-\mathrm{down}-\mathrm{regulated}\right)}{\sqrt{\mathrm{total}}\mathrm{DEGs}}$) and is displayed coded by color. Relative gene expression levels of the GO terms **c** “white fat cell differentiation” and **d** “regulation of inflammatory response” are presented in heat maps. Expression levels of **e** *Pparg*, **f** *Tnf*, **g** *Il1b*, and **h** *Il6* were validated using RT-PCR in fat grafts at weeks 1, 4, and 8 from old and young donors. Data are presented as the mean ± SD (n = 6 per group)

Among 25,940 detected genes, 367 were significantly upregulated and 150 were downregulated (padj < 0.05 and log2 (fold change) > 1) in the graft tissue from old donors compared with grafts from young donors (Fig. 6a and Supplemental Table 2). The 517 differentially expressed genes (DEGs) were used for GO enrichment analysis; 553 significantly enriched (padj < 0.01) GO biological processes were identified. GO terms associated with adipogenesis including “white fat cell differentiation,” “brown fat cell differentiation,” “fat cell differentiation,” “regulation of fat cell differentiation,” and “positive regulation of fat cell differentiation” were significantly decreased in the O-O group compared with the Y-O group (Fig. 6b). In addition, genes relating to “white fat cell differentiation” including *Adig*, *Scd1*, *Fgf10*, *Pparg*, and *Fabp4* were expressed at a significantly lower level in the O-O group than in the Y-O group (Fig. 6c).

These results suggest that adipogenesis was induced in grafts from young donors as early as 1 week after grafting and was suppressed in grafts from old donors.

### Grafts from old donors display a proinflammatory phenotype at week 1

RNA-seq analysis revealed that GO terms associated with tissue inflammation including “monocyte chemotactic protein-1 production,” “interleukin-17 production,” “chronic inflammatory response,” “acute inflammatory response,” “chemokine-mediated signaling pathway,” “tumor necrosis factor production,” “interferon-gamma production,” “interleukin-6 production,” “regulation of chemotaxis,” “interleukin-1 beta production,” “regulation of inflammatory response,” “cytokine secretion,” “cytokine-mediated signaling pathway,” and “chemotaxis” were significantly enriched and increased in the O-O group compared with the Y-O group (Fig. 6b). Notably, a group of genes related to the “regulation of inflammatory response” including *Per1*, *Ppard*, *Tgm2*, *Tnfrsf1b*, *Zfp36*, *Nt5e*, *Casp4*, *Nod2*, *Rabgef1*, *Adora2b*, *Tnip1*, *Pglyrp1*, *Tlr2*, *Cd24a*, *Il2ra*, *Ccr7*, *Tnfsf11*, *Socs3*, *Mefv*, *Nfkbia*, *Tarm1*, *Siglece*, *Nlrp12*, *Tnf*, *Tnfaip3*, *Nos2*, *Ptgs2*, *Il6*, *Ccl3*, *Ccl4*, *Nlrp3*, *S100a8*, *S100a9*, *Il1b*, and *Acod1* were significantly upregulated in the O-O group compared with the Y-O group (Fig. 6d). These results suggested that grafting fat from aged donors resulted in a proinflammatory response that was closely related to impaired adipogenesis.

To validate this result, we performed RT-PCR analysis for the proadipogenic gene *Pparg* and the proinflammatory cytokine genes *Tnf*, *Il1b*, and *Il6* in samples at 1, 4, and 8 weeks after fat grafting in the O-O and Y-O groups. In line with the impaired adipogenesis found in grafts from the O-O group, *Pparg* was expressed at significantly lower levels in the O-O group than in the Y-O group at all time points (Fig. 6e). Similar to the RNA-seq results, expression of *Tnf*, *Il1b*, and *Il6* was significantly higher in the O-O group than in the Y-O group at 1 and 4 weeks post-grafting. *Il1b* was expressed at significantly higher levels in the O-O group than in the Y-O group at week 8 (Fig. 6f–h).

### Donor age negatively impacts differentiation of AD-MSCs

**Fig. 7:**
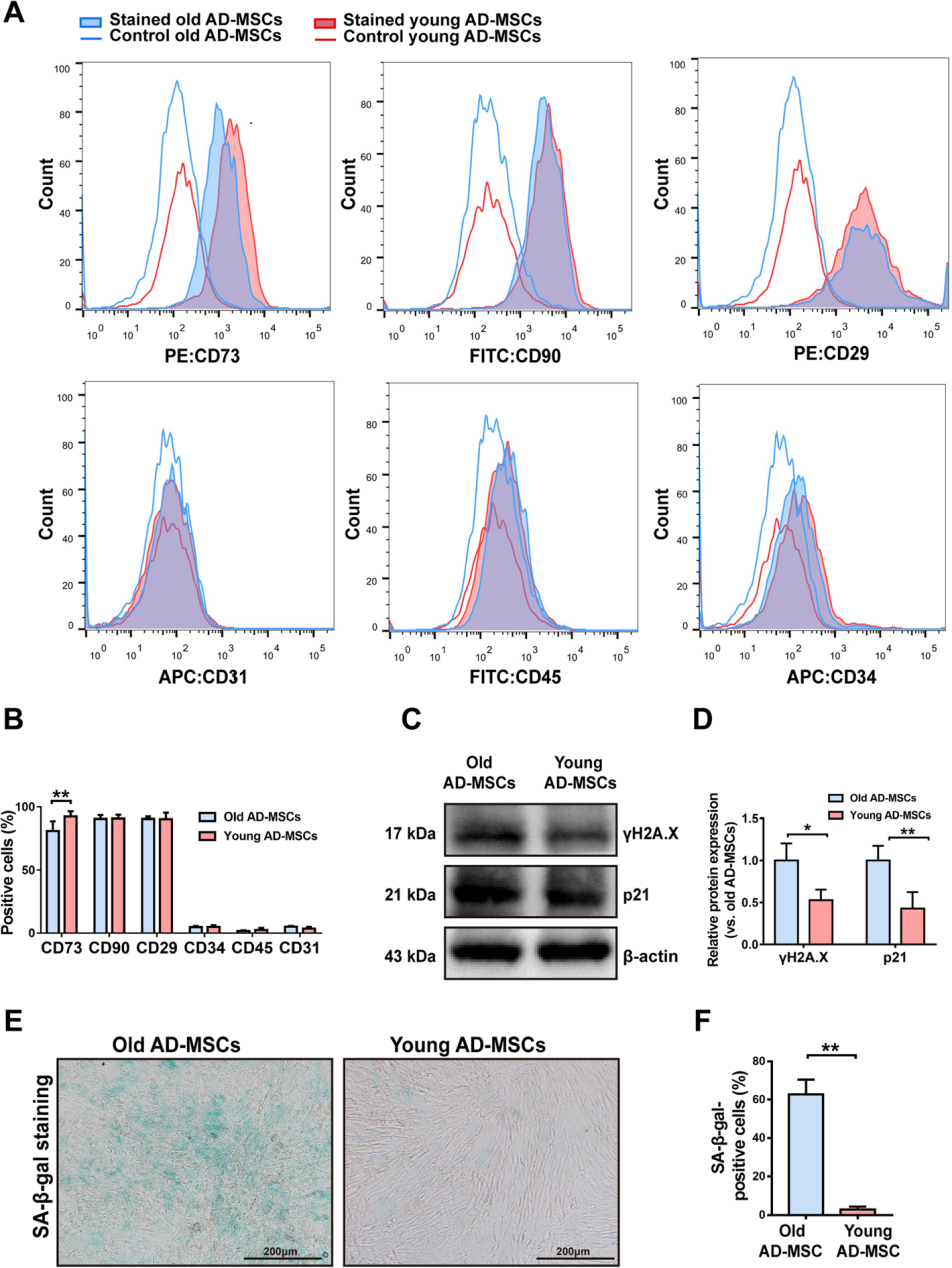
Characterization of old and young AD-MSCs. **a**, **b** Flow cytometric analysis of antigen expression on the surface of old and young AD-MSCs. **c**, **d** Western blot analyses of p21 and γH2A.X proteins in old and young AD-MSCs. **e**, **f** SA-β-gal staining and quantification of SA-β-gal-positive cells among old and young AD-MSCs. **p < 0.01 and *p < 0.05 vs. data from different groups. Data are presented as the mean ± SEM of three independent experiments

Considering that new mature adipocytes are mostly derived from AD-MSCs, which are an admixture of donor and recipient cells, during fat graft regeneration, we further explored the potential mechanisms underlying the role of age in regulation of AD-MSC differentiation during fat graft regeneration in the O-O and Y-O groups. We first analyzed the surface markers of young and old AD-MSCs at passage 3 by flow cytometry. Both groups of cells exhibited the classical pattern of AD-MSC marker expression. Although a significantly higher percentage of young AD-MSCs than old AD-MSCs was CD73-positive, the percentages of cells positive for CD29 and CD90 did not significantly differ between the two groups. Both young and old AD-MSCs were negative for the AD-MSC markers CD31, CD45, and CD34 (Fig. 7a, b). AD-MSCs obtained from old donors displayed senescent features, including increased expression of p21 and γH2A.X proteins (Fig. 7c, d) and elevated SA-β-gal activity (Fig. 7e, f).

**Fig. 8:**
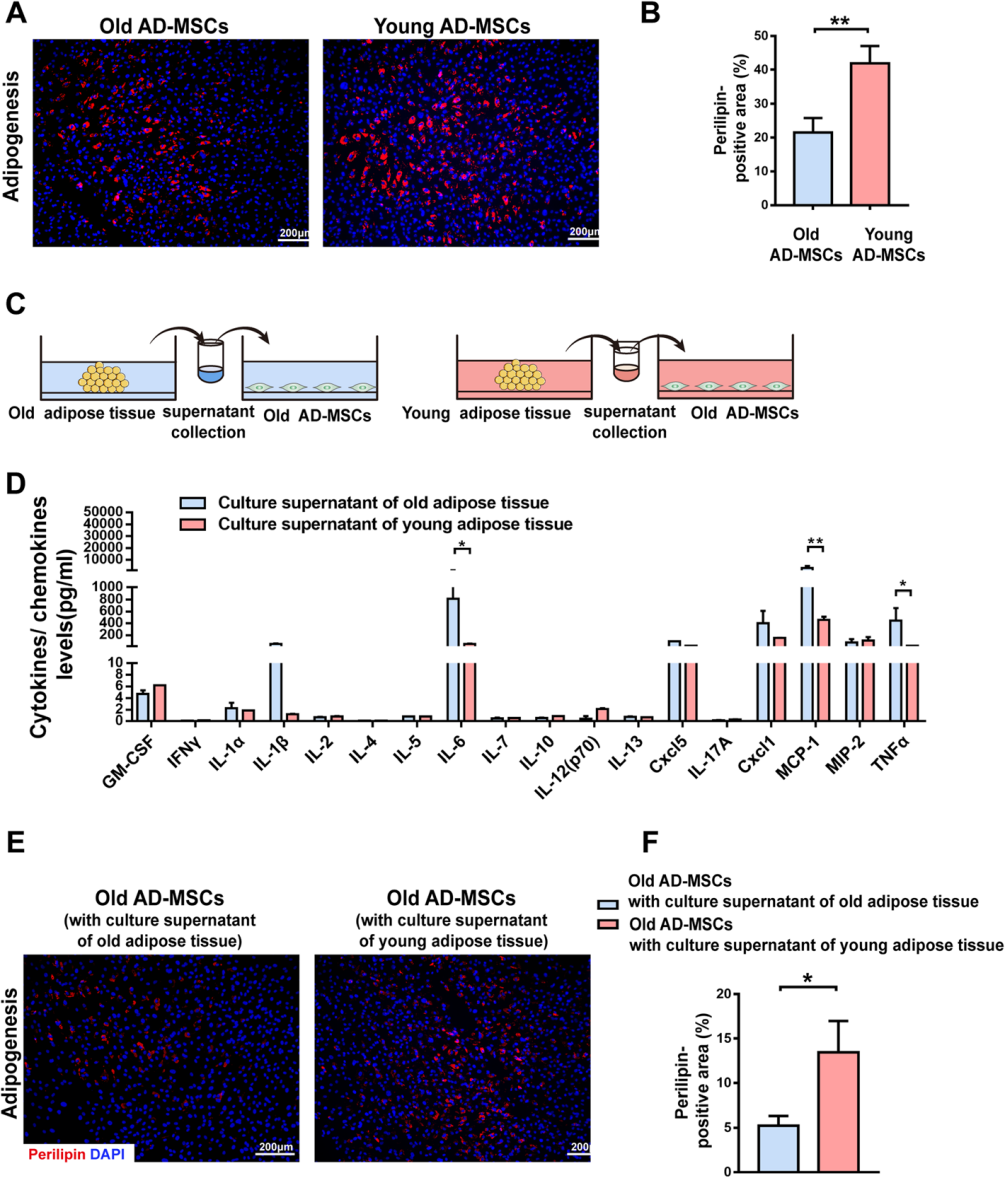
Donor age negatively impacts differentiation of AD-MSCs. **a**, **b** Perilipin staining and quantification of the perilipin-positive area in old and young AD-MSCs. **c** Illustration of the co-culture model. **d** MILLIPLEX® MAP assays measuring the levels of 18 cytokines/chemokines in conditioned media of old and young adipose tissue. **e**, **f** Perilipin staining and quantification of the perilipin-positive area in old AD-MSCs treated with the culture supernatant of old or young adipose tissue. **p < 0.01 and *p < 0.05 vs. data from different groups. Data are presented as the mean ± SEM of three independent experiments

Adipogenic differentiation was assessed by staining cells for perilipin to label the cytoplasmic accumulation of lipids/triglycerides after 15 days of differentiation. Compared with AD-MSCs isolated from young donors, AD-MSCs obtained from old donors had a significantly reduced adipogenic differentiation potential (Fig. 8a, b). In addition, the ability of conditioned media of old and young adipose tissue cultures to modulate adipogenic differentiation of AD-MSCs from old recipients was investigated (Fig. 8c). The levels of 18 cytokines/chemokines in the culture supernatant of adipose tissue were measured using MILLIPLEX® MAP assays. The levels of some SASP factors, including IL-6, MCP-1, and TNF-α, were significantly higher in the culture supernatant of old adipose tissue than in the culture supernatant of young adipose tissue (Fig. 8d). Furthermore, immunofluorescence staining demonstrated that the perilipin-positive area of differentiated old AD-MSCs was markedly decreased by treatment with the culture supernatant of old adipose tissue, but increased by treatment with the culture supernatant of young adipose tissue (Fig. 8e, f).

## Discussion

This study demonstrates that age had a detrimental effect on fat graft outcomes. Grafts harvested from and grafted into older mice are smaller and of a poorer quality than grafts harvested from and grafted into young mice. Donor age has a greater effect on graft outcomes than recipient age, as indicated by reduced adipogenesis and elevated expression of SASP factors, especially IL-6 and TNF-α. Furthermore, we demonstrated that donor age negatively impacted the adipogenic differentiation potential of AD-MSCs in vitro, and the culture supernatant of old donor fat tissue significantly decreased the adipogenic differentiation ability of AD-MSCs of old recipients compared with the culture supernatant of young donor fat tissue.

Regarding the optimal timing of fat grafting, some surgeons suggest that beginning soft-tissue reconstruction with fat grafting in patients at an earlier age may be preferable to late correction [22, 23]. In a study comparing the outcomes of serial fat grafting to correct soft-tissue deficiency with microvascular free tissue transfer for craniofacial microsomia, Tanna and colleagues reported that fat grafting can be safely performed in combination with other operative interventions throughout childhood and can result in good symmetry when performed before the skeletal deficiency is addressed [24]. Our study demonstrated that fat graft volume was significantly smaller and adipogenesis in grafts was lower in the O-O group than in the Y-Y group. This suggests that fat grafts are smaller and of a poorer quality in old adults than in young adults and that age may be a risk factor for adverse outcomes of fat grafting.

Another important finding of this study is that graft outcomes were affected more by donor age than by recipient age. After 12 weeks, the volume of fat grafts harvested from old or young donors did not significantly differ between old and young recipients (O-O vs. O-Y, *p* > 0.05; Y-O vs. Y-Y, *p* > 0.05), but was smaller when fat grafts from old donors were used (O-O vs. Y-O, *p* < 0.05; O-Y vs. Y-Y, *p* < 0.05). As expected, fat graft regeneration was observed in all four groups consistent with the “cell replacement theory,” which states that grafted fat can be categorized into three zones from the periphery to the center, namely, the survival, regeneration, and necrosis zones. After fat grafting, most adipocytes undergo necrosis and are subsequently replaced by regeneration of adipocytes [8, 25]. Similarly, our study confirmed that necrotic adipocytes were replaced by newly generated cells after fat grafting, as evidenced by the significantly increased area of perilipin-positive adipocytes from week 4 to 12 in all four groups. However, adipogenesis in grafts from older donors was incomplete, which was characterized by smaller adipocytes and larger undifferentiated regions. This finding is in agreement with other studies demonstrating that donor environment impacts fat graft outcomes more than recipient sites. For instance, fat depots excised from one part of the animal and grafted to another location (subcutaneous fat tissue grafted into the visceral cavity), or grafts harvested from low-estrogenic conditions into a normal estrogen environment, resemble the pre-graft characteristics after remodeling [26–28]. In addition, by performing RNA-seq and RT-PCR analysis to investigate age-related changes in the transcriptome of fat tissue from old and young mice as well as fat grafts from the O-O and Y-O groups, we found that the detrimental effects of old donors on fat graft regeneration may be attributable to impaired adipogenesis and increased expression of SASP factors.

The mechanisms underlying the inhibitory effect of aged donors on adipogenesis in fat grafts need to be elucidated. AD-MSCs are the main cell population that contributes to regeneration of adipocytes in all types of adipose tissue remodeling/expansion, such as developmental growth, hyperplasia in obesity, and repair processes after injury/ischemia [29, 30]. A substantial amount of evidence supports the therapeutic use of AD-MSCs to improve long-term graft retention [31]. The overall volume of a fat graft reportedly depends on the degree of survival in the regenerating zone, which contains AD-MSCs with the potential to differentiate and replace adipocytes lost in the necrotic zone [9, 32]. Thus, surviving donor AD-MSCs may partly contribute to subsequent adipogenesis. In vitro, we found that AD-MSCs isolated from old donors displayed senescent features and had a reduced adipogenic differentiation potential compared with AD-MSCs isolated from young donors. Thus, we propose that impaired adipogenesis observed in fat grafts from old donors may be partly attributable to a cell-intrinsic defect in the regenerative capacity of aged AD-MSCs.

The quality of the macroenvironmental niche into which AD-MSCs are placed may be of similar importance as the senescence of AD-MSCs for long-term fat graft outcomes. Tissue remodeling in grafted fat is initiated by zonal necrosis of adipocytes, which triggers activation of AD-MSCs and infiltration of recipient cells. On the other hand, some stem/progenitor cells derived from recipient bone marrow (bone marrow-derived mesenchymal stem cells, BM-MSCs) may also contribute to fat graft remodeling after grafting [9]. However, the role of BM-MSCs in fat graft remodeling is unclear and must be investigated further. AD-MSCs derived from recipients also reportedly contribute to generation of adipocytes in fat grafts [10]. Aged adipose tissue has been reported to release proinflammatory cytokines that impair differentiation of AD-MSCs necessary for regeneration [16]. Senescent cells are characterized by upregulation of proinflammatory cytokines, chemokines, and proteases, which are termed SASP factors [33]. Given the complex nature of the SASP, senescent cells impact various biological processes that involve paracrine signaling including inflammation [34], wound healing [35], and other types of tissue repair [36]. In this study, several SASP factors, including IL-6, IL-1β, and TNF-α, were highly expressed during the beginning and later phases of regeneration in fat grafts from old donors. It is plausible that SASP factors secreted from senescent cells in old donors induce inflammatory responses in fat grafts. Although several studies have reported that an acute inflammatory response promotes extracellular matrix remodeling and angiogenesis, which benefits adipogenesis [25, 37], sustained high proinflammatory cytokine expression in adipose tissue is associated with inhibition of adipogenesis in fat grafts [32, 38–40]. Finally, in vitro experiments showed that the levels of some SASP factors, including IL-6, MCP-1, and TNF-α, were significantly higher in the culture supernatant of old adipose tissue than in the culture supernatant of young adipose tissue, which significantly impaired the adipogenic differentiation ability of AD-MSCs derived from old recipients. Consistent with our study, type 2 diabetes mellitus, obesity, and insulin resistance result in persistent production of proinflammatory cytokines such as TNF-α, IL-1β, and IL-6, which typically inhibit adipogenesis [40].

In addition to the major SASP factors mentioned above, genes related to other SASP factors including *Mmp11*, *Serpine1*, and *Il17* were also upregulated in fat tissue from old mice. Matrix metalloproteinases (MMPs) and SERPINE1 (also known as PAI-1) secreted by senescent cells contribute to the development of tissue fibrosis [41, 42]. Furthermore, MMP-11 and IL-17 reportedly negatively regulate adipogenesis by reducing pre-adipocyte differentiation and reversing mature adipocyte differentiatio n[43–45]. Based on our findings, we propose that the release of SASP factors in fat grafts from old donors also leads to anti-adipogenic effects on AD-MSCs of recipients.

## Conclusions

Overall, this study demonstrated that age has detrimental effects on fat graft outcomes by suppressing adipogenesis of AD-MSCs and upregulating expression of SASP factors, and graft outcomes are affected more by donor age than by recipient age. Thus, future studies that aim to rejuvenate fat grafts from old donors are required. Given the successful fat tissue regeneration found in grafts from younger donors, approaches for the old adults could focus on banking younger adipose tissue for later use. To do this, a previously reported cryopreservation protocol may be utilized, which allows the attainment of a nearly normal fat graft appearance after cryopreservation when compared with fresh fat grafts [46–48]. Preservation of adipose tissue at a younger age, when biological activity is greatest, could be ideal for future regenerative medicine applications.

## Supplementary Information

**Additional file 1: Supplemental Table 1.** All identified DEGs between fat tissue from old and young mice.

**Additional file 2: Supplemental Table 2.** All identified DEGs between fat grafts in the O-O and Y-O groups at week 1.

## Data Availability

All data generated or analyzed in this study are included in this article.

## Abbreviations

AD-MSC: Adipose-derived mesenchymal stem cell
BM-MSC: Bone marrow-derived mesenchymal stem cell
Cxcl1: Chemokine (C-X-C motif) ligand 1
DEG: Differentially expressed gene
FPKM: Fragments per kilobase of transcript per million fragments mapped
GM-CSF: Granulocyte-macrophage colony-stimulating factor
GO: Gene Ontology
HE: Hematoxylin and eosin
IFNγ: Interferon γ
IL: Interleukin
MCP: Monocyte chemoattractant protein
MFI: Median fluorescence intensity
MIP: Macrophage inflammatory protein
MMP: Matrix metalloproteinase
PBS: Phosphate-buffered saline
RNA-seq: RNA sequencing
RT-PCR: Real-time polymerase chain reaction
SA-β-gal: Senescence-associated β-galactosidase
SASP: Senescence-associated secretory phenotype
SEM: Standard error of mean
TBS: Tris-buffered saline
TNF: Tumor necrosis factor
γH2A.X: Histone H2AX phosphorylation
